# Spread of Vaccinia Virus to Cattle Herds, Argentina, 2011

**DOI:** 10.3201/eid2009.140154

**Published:** 2014-09

**Authors:** Ana Paula Moreira Franco-Luiz, Alexandre Fagundes-Pereira, Galileu Barbosa Costa, Pedro Augusto Alves, Danilo Bretas Oliveira, Cláudio Antônio Bonjardim, Paulo César Peregrino Ferreira, Giliane de Souza Trindade, Carlos Javier Panei, Cecilia Mónica Galosi, Jônatas Santos Abrahão, Erna Geessien Kroon

**Affiliations:** Universidade Federal de Minas Gerais, Belo Horizonte, Brazil (A.P.M. Franco-Luiz, A. Fagundes-Pereira, G.B. Costa, P.A. Alves, D.B. Oliveira, C.A. Bonjardim, P.C.P. Ferreira, G.S. Trindade, J.S. Abrahão, E.G. Kroon);; Universidad Nacional de la Plata, Buenos Aires, Argentina (C.J. Panei, C.M. Galosi);; Consejo Nacional Investigaciones Científicas y Técnicas, Buenos Aires (C.J. Panei);; Comisión de Investigaciones Científicas Provincia de Buenos Aires,, Buenos Aires (C.M. Galosi)

**Keywords:** vaccinia virus, orthopoxvirus, bovine herds, cattle herds, poxvirus, Argentina, viruses, exanthematous lesions, cattle, humans, beef producers, milk producers, veterinary surveillance

**To the Editor:** Since 1999, several zoonotic outbreaks of vaccinia virus (VACV) infection have been reported in cattle and humans in rural areas of Brazil. The infections have caused exanthematous lesions on cows and persons who milk them, and thus are detrimental to the milk industry and public health services (*1*,*2*). In Brazil during the last decade, VACV outbreaks have been detected from the north to the extreme south of the country (*1*–*4*). Because Brazil shares extensive boundaries with other South American countries, humans and cattle on dairy and beef-producing farms in those countries may be at risk of exposure to VACV. To determine if VACV has spread from Brazil to Argentina, we investigated the presence of VACV in serum samples from cattle in Argentina.

During 2011, we obtained serum samples from 100 animals (50 dairy and 50 beef cattle) on farms in Córdoba, Corrientes, Entre Ríos, and Santa Fe Provinces in Argentina (Technical Appendix, panel A). No VACV cases had been reported in humans or cattle in these provinces. However, Corrientes Province borders the Brazilian state of Rio Grande do Sul, where VACVs (Pelotas 1 and Pelotas 2 viruses) were isolated during an outbreak affecting horses in 2008 (*2*).

To determine the presence of neutralizing antibodies in the serum samples, we used an orthopoxvirus 70% plaque-reduction neutralization test as described (*4*). On the basis of previous studies that detected viral DNA in serum samples (*4*–*6*), we used real-time PCR to amplify the highly conserved orthopoxvirus vaccinia growth factor (*vgf*) gene DNA (P.A. Alves, unpub. methods).

To amplify the hemagglutinin (HA) gene DNA from the serum samples, we used real-time PCR with primers as described by de Souza Trindade et al. 2008 (*7*). The HA PCR products were directly sequenced in both orientations by using specific primers and capillary electrophoresis (Genetic Analyzer 3130; Applied Biosystems, Grand Island, NY, USA). We used ClustalW (http://www.clustal.org) and MEGA4 software (http://megasoftware.net/) to align nucleotide sequences and construct a phylogenetic tree (neighbor-joining method, 1,000 bootstraps) from the obtained HA fragment.

Of the 50 dairy cattle samples, 4 (8.0%) had neutralizing antibodies against orthopoxvirus; of these, 3 (75.0%) had titers of 100 neutralizing units (NU)/mL, and 1 (25.0%) had a titer of 400 NU/mL. Of the 50 beef cattle, 8 (16.0%) had antibodies to orthopoxvirus, 1 (12.5%) of which had a titer of 800 NU/mL. Most of the positive samples were from cattle in Corrientes and Entre Ríos Provinces (Table).

**Table:** Diagnosis of *Orthopoxvirus* infection in beef and dairy cattle during a study of the spread of vaccinia virus to cattle herds, Argentina, 2011*

Province	Cattle type	No. farms sampled	No. serum samples tested	No. positive samples, by level of NU/mL against orthopoxvirus†						No. (%) positive by real-time PCR	
				100	200	400	800	Total (%)		*vgf*	HA
Córdoba	Dairy	1	25	0	0	1	0	1 (4.0)		2 (8.0)	0
Santa Fe	Dairy	1	25	3	0	0	0	3 (12.0)		0	0
Corrientes	Beef	>2‡	8	0	1	1	0	2 (25.0)		1 (12,5)§	1 (12,5)§
Entre Ríos	Beef	5	42	2	2	1	1	6 (14.3)		2 (4,8)	1 (2,4)§
Total	Dairy and beef	>9	100	5	3	3	1	12 (12.0)		5 (5.0)	2 (2.0)

*HA, hemagglutinin gene DNA; NU, serum dilution at which 70% plaque reduction per mL occurs; *vgf*, orthopoxvirus vaccinia growth factor gene DNA. †Determined by plaque-reduction neutralization test. ‡Samples were obtained from several farms in Corrientes Province. §Animal was also positive by plaque-reduction neutralization test.

Of the 100 serum samples, 5 (3 from beef and 2 from dairy cattle) were positive for *vgf* by real-time PCR. HA DNA was amplified from 2 of the 3 *vgf* PCR–positive beef cattle samples; plaque-reduction neutralization test results were also positive for the 2 samples (Table). 

Alignment of the HA fragment nucleotide sequence of the isolates from Argentina showed that the sequence was highly similar to that of the homologous gene of VACV isolates from Brazil. Furthermore, the sequences showed a signature deletion that is also present in the sequences of VACV isolates from Brazil. Compared with sequences for other VACV isolates, those from Argentina had 2 polymorphisms (Technical Appendix, panel C). The HA sequences from the isolates from Argentina demonstrated 100% identity among themselves and exhibited higher identity with group 1 (98.2% identity) versus group 2 (93.6% identity) isolates from Brazil (Technical Appendix, panel D). In the phylogenetic tree based on the HA nucleotide sequences (Technical Appendix, panel B), the VACVs from Argentina clustered with several group 1 VACVs detected during outbreaks in Brazil.

Although no outbreaks of exanthematous VACV infection have been described in cattle or humans in Argentina, we detected neutralizing antibodies against orthopoxvirus and detected VACV DNA in serum samples from cattle in the country. Most of the seropositive samples were from cattle in Entre Ríos Province, which shares a border with Uruguay, and Corrientes Province, which shares a border with Rio Grande do Sul State in Brazil, where Pelotas VACVs have been isolated (*2*). 

We believe that the seropositive cattle in this study may have been exposed to VACV, the only orthopoxvirus known to be circulating in South America (*1*–*4*,*8*–*10*). Despite veterinary surveillance efforts of border control organizations, VACV control may be hampered by the circulation of infected rural workers and the misdiagnosis of VACV infection; misdiagnoses occur because VACV lesions resemble those of other exanthematous diseases. Moreover, peridomestic rodents have been hypothesized to act as VACV hosts, and could facilitate the spread of VACV in border areas (*10*). In addition, we could not rule out the circulation of autochthonous VACV in Argentina, but this is a less likely explanation. Our findings suggest that cattle herds in areas of Argentina near the border with Brazil may be exposed to VACV from Brazil and, thus, may be at risk for VACV infection. Further research is needed to determine the risk factors for VACV infection and to assess the circulation of VACV in South America

Technical Appendix Map of Argentina, indicating locations where blood samples were collected from cattle and results of phylogenetic analysis of vaccine virus isolated from the samples.
