# Supplementary material for: Spread of Vaccinia Virus to Cattle Herds, Argentina, 2011
Source: Emerg Infect Dis. 2014 Sep;20(9):1576–8. doi: 10.3201/eid2009.140154 (PMC4178398; doi:10.3201/eid2009.140154)
Supplement: Technical Appendix — Map of Argentina, indicating locations where blood samples were collected from cattle and results of phylogenetic analysis of vaccine virus isolated from the samples. [file 14-0154-Techapp-s1.pdf]

Article DOI: http://dx.doi.org/10.3201/eid2009.140154

Spread of Vaccinia Virus to Cattle Herds, Argentina, 2011

Technical Appendix

A

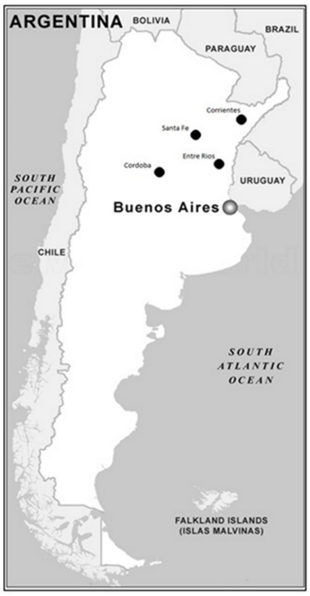

B

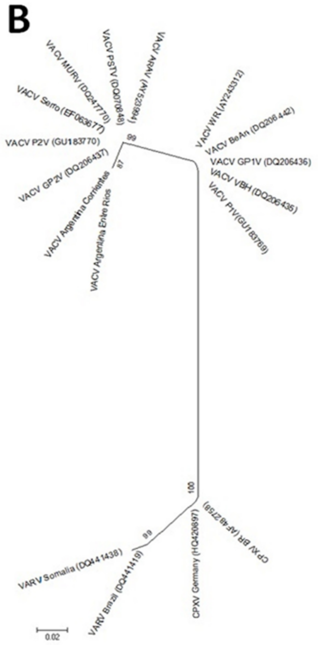

C

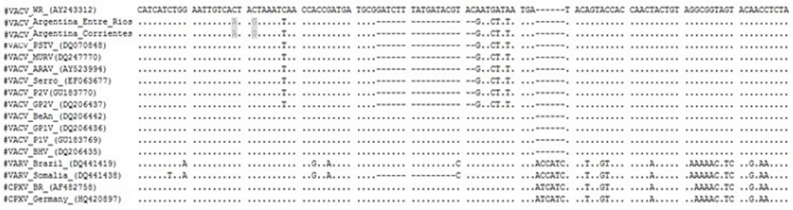

D

	VACV WR	VACV Argentina Entre Rios	VACV Argentina Corrientes	VACV PSTV	VACV MURV	VACV ARAV	VACV Serro	VACV P2V	VACV GP2V	VACV BeAn	VACV GP1V	VACV P1V	VACV BHV	VARV Brazil	VARV Somalia	CPXV BR	CPXV Germany
VACV WR (AY243312)																	
VACV Argentina Entre Rios	93,6																
VACV Argentina Corrientes	93,6	100															
VACV PSTV (DQ070848)	95,5	98,2	98,2														
VACV MURV (DQ247770)	95,5	98,2	98,2	100													
VACV ARAV (AY523994)	95,5	98,2	98,2	100	100												
VACV Serro (EF063677)	95,5	98,2	98,2	100	100	100											
VACV P2V (GU183770)	95,5	98,2	98,2	100	100	100	100										
VACV GP2V (DQ206437)	95,5	98,2	98,2	100	100	100	100	100									
VACV BeAn (DQ206442)	100	93,6	93,6	95,5	95,5	95,5	95,5	95,5	95,5								
VACV GP1V (DQ206436)	100	93,6	93,6	95,5	95,5	95,5	95,5	95,5	95,5	100							
VACV P1V (GU183769)	100	93,6	93,6	95,5	95,5	95,5	95,5	95,5	95,5	100	100						
VACV BHV (DQ206435)	100	93,6	93,6	95,5	95,5	95,5	95,5	95,5	95,5	100	100	100					
VARV_Brazil(DQ441419)	78,2	71,8	71,8	73,6	73,6	73,6	73,6	73,6	73,6	78,2	78,2	78,2	78,2				
VARV Somalia (DQ441438)	77,3	70,9	70,9	72,7	72,7	72,7	72,7	72,7	72,7	77,3	77,3	77,3	77,3	99,1			
CPXV BR (AF482758)	81,8	75,5	75,5	77,3	77,3	77,3	77,3	77,3	77,3	81,8	81,8	81,8	81,8	96,4	95,5		
CPXV Germany (HQ420897)	81,8	75,5	75,5	77,3	77,3	77,3	77,3	77,3	77,3	81,8	81,8	81,8	81,8	96,4	95,5	100	

Technical Appendix Figure. A) Map with black dots indicating the 4 provinces where blood samples were collected for the study of the spread of vaccinia virus (VACV) to cattle herds, Argentina, 2011. B) Phylogenetic tree based on nucleotide sequences of orthopoxvirus hemagglutinin (HA) gene. The tree was constructed with HA sequences by using the neighbor-joining method with 1,000 bootstrap replicates and the Tamura 3-parameter model in MEGA4 (http://megasoftware.net/). Bootstrap values are represented on branches and only values >90% are shown. Nucleotide sequences were obtained from GenBank. Scale bar represents 0.02 substitutions per nucleotide position. C) Nucleotide sequence of the VACV Argentina A56R (HA) gene compared with homologous sequences from several other orthopoxviruses. The samples from Argentina showed 2 polymorphisms (C–A and C–G) in comparison with other VACV isolates. D) A56R (HA) gene nucleotide identity matrix. The analysis involved 17 nt sequences. Evolutionary analyses were conducted in MEGA4.
